# N-Glycosylation on Asn50 of SND1 Is Required for Glioma U87 Cell Proliferation and Metastasis

**DOI:** 10.1155/2022/5239006

**Published:** 2022-09-29

**Authors:** Ying Zhou, Qingyu Li, Jianfeng Zheng, Nengming Lin

**Affiliations:** ^1^Department of Clinical Pharmacology, Key Laboratory of Clinical Cancer Pharmacology and Toxicology Research of Zhejiang Province, Affiliated Hangzhou First People's Hospital, Cancer Center, Zhejiang University School of Medicine, Hangzhou, Zhejiang 310006, China; ^2^Department of Clinical Pharmacy, Affiliated Hangzhou First People's Hospital, Zhejiang University School of Medicine, Hangzhou, Zhejiang 310006, China; ^3^The Fourth School of Clinical Medicine, Zhejiang Chinese Medical University, Hangzhou, Zhejiang 310053, China; ^4^Department of Obstetrics and Gynecology, Affiliated Hangzhou Hospital, Nanjing Medical University, Hangzhou, Zhejiang 310008, China

## Abstract

Staphylococcal nuclease domain-containing protein 1 (SND1) is an evolutionarily conserved multidomain protein, which has gained attention recently due to its positive regulation in several cancer progression and metastatic spread. However, the specific contribution of SND1 glycosylation in glioma remains uncertain. In the current study, we confirmed that SND1 was highly expressed in human glioma. Using site-directed mutagenesis, we created four predicted N-glycosylation site mutants for SND1 and provided the first evidence that SND1 undergoes N-glycosylation on its Asn50, Asn168, Asn283, and Asn416 residues in human glioma U87 cells. In addition, we found that removing the N-glycans on the Asn50 site destabilized SND1 and led to its endoplasmic reticulum-associated degradation. Furthermore, destabilized SND1 inhibits the glioma cell proliferation and metastasis. Collectively, our results reveal that N-glycosylation at Asn50 is essential for SND1 folding and trafficking, thus essential for the glioma process, providing new insights for SND1 as a potential disease biomarker for glioma.

## 1. Introduction

Glioma is one of the most common cancers that occurred in the central nervous system, which is considered essentially incurable [1, 2]. Even after maximal safe resection combining with radiochemotherapy as well as specific anticancer regent temozolomide treatment, the median survival account for glioma is still less than two years [3]. In the 2007 WHO guidelines, glioma was classified into several subtypes according to the appearance of original tissues as well as the histologic criteria. The malignancy degree was classified into grades II–IV based on the morphological criteria [4]. Among the different types of gliomas, glioblastoma (GBM), the representative of grade IV gliomas, occupied more than half of the incidence, which is the most malignant and notorious for drug therapy resistance [4]. In addition to drug resistance, most of the agents that directed against the GBMs specific targets have been proved to be failed [5]. Although numerous efforts have been achieved in the past decades, about 50% GBM patients could not survive even in the first year after diagnosis [6]. The overall 5-year survival rate was reported less than 5%, and this situation is even worse in elderly patients [7]. Compared with high-grade gliomas, low-grade gliomas (LGG) (grades II and III) have a less frequent occurrence. They were likewise less aggressive and show a better prognosis. However, outcomes of LGG are still fatal in most patients due to the high disabling morbidity after treatment [8]. These frustrating clinical results make glioma an urgent topic in cancer research. It is of great clinical significance to discovery hallmarks that could target for glioma therapy.

Glycosylation is one of the most elemental forms of protein modification in mammalian multicellular cells [9]. The major kinds of glycosylation were classified including N-, O-, and C-linked glycosylation, glypiation, and phosphoglycosylation. Among them, N-glycosylation was the most common type occupying more than half of the glycosylated proteins [10]. The procedure of N-glycosylation is in fact a process that genes undergo the nontemplated translation and then transfer into the endoplasmic reticulum (ER) followed by the Golgi apparatus for trimming, though the purpose for trimming is different [11, 12]. In this regard, epigenetic regulation of genes involved in protein glycosylation is part of the mechanisms in which research is still scarce.

To date, more and more studies have demonstrated that an altered N-glycosylation profile is closely linked to the occurrence and promotion of cancer progression [13]. In glioma, glycosylation defects were reported to promote glioma cells invasion and migration [14, 15]. However, the relevant studies are still scarce, and the regulatory mechanism and the molecular basis for this N-glycosylation alteration-induced glioma pathology are still not fully found. To gain further insight into the involvement of protein N-glycosylation in glioma is important for elucidating the pathogenesis and identifying new therapeutic targets for glioma.

Staphylococcal nuclease domain-containing protein 1 (SND1), also known as Tudor staphylococcal nuclease (TSN), is a multidomain protein that composes four staphylococcal nuclease (SN) domains (SN1-4) as well as a fusion of Tudor and partial SN domain (TSN5) [16]. It is one of the protein members of oligonucleotide/oligosaccharide binding-fold (OB-fold) superfamily that binds to DNA/RNA by the *β*-barrel of OB-fold [17]. According to the structure of SND1, SN domains, especially SN3-4, showed nuclease activity and the ability of RNA binding. At the same time, TSN5 in the C-terminal was reported to interact with methylated Lys/Arg ligands. SND1 could regulate RNA splicing and editing as both the nuclease and the ligand [18]. It also participated in the RNA-induced silencing complex (RISC) that is involved in the miRNA-mediated silencing [19, 20]. Quite apart from that, among the OB-fold superfamily, SND1 is the rare member that participates in the interaction of multifarious proteins [21]. Recent report suggested SND1 functions as a MTDH-interacting protein which was occupied in the extended protein groove between SN1 and SN2 domains by MTDH-specific peptide motif in breast cancer [21]. Clinical and experimental studies also indicate that high expression of SND1 was observed in a spectrum of cancers including glioma, prostate cancer, lung cancer, and liver cancer [22–26]. It seems that SND1 is a rare gene that acts in almost the virtually gene expression processes, ranging from gene transcription, mRNA splicing, to RNA silencing and protein modification. However, the functional role of N-glycosylation of SND1 in glioma remains to be determined.

In this study, we suggest that SND1 is highly expressed in the glioma. And we confirm that the Asn50, Asn168, Asn283, and Asn416 of SND1 are all occupied by N-glycans. We demonstrate that the loss of the N-glycosylation at Asn50 site leads to a mislocalization of SND1 in ER and blocks the SND1 transfer into Golgi. Last but not the least, we provide the experimental evidence of the deletion of N-glycans on the Asn50 of SND1 in charge of cell proliferation and metastasis.

## 2. Materials and Methods

### 2.1. Reagents and Antibodies

Anti-Tudor-SN (SND1, sc-166676), anti-GFP (sc-9996), anti-calnexin (CNX, sc-46669), and anti-GAPDH (sc-47724) were purchased from Santa Cruz Biotechnology (Santa Cruz, CA, USA). Anti-GRP78 (AF0171) was purchased from Beyotime Biotechnology (Shanghai, China). Anti-CHOP (2895S) and anti-GRP94 (20292S) were purchased from Cell Signaling Technology, Inc. (Danvers, MA, USA). Anti-GM130 (11308-1-AP) was purchased from ProteinTech (Rosemont, USA). MG-132 (C_26_H_41_N_3_O_5_, 1211877-36-9) was purchased from the Selleck.cn (Shanghai, China). Chloroquine (C_18_H_26_C_l_N_3_, 54-05-7) was purchased from the Topscience Co. Ltd (Shanghai, China). Geneticin (G418, C_20_H_40_N_4_O_10_∙2H_2_SO_4_, 1150GR001) was purchased from the BioFroxx (Guangzhou, China). PNGase F (#P0704L) was purchased from the New England Biolabs Inc. (Ipswich, MA, USA). Tunicamycin (TM) was purchased from Beyotime Biotechnology (Shanghai, China).

### 2.2. Cell Culture and Treatment

Human glioma U87 cells were purchased from the Chinese Academy of Sciences (Shanghai, China). Cells were cultured in 37°C by DMEM containing 10% FBS purchased from Gibco (Grand Island, NY, USA). To completely inhibit the formation of N-glycans in glioma cells, 1 *μ*g/mL TM was added into the culture medium for 48 h [27]. To cleave the innermost GlcNAc and attached N-glycans from asparagine residues, cells were collected and washed by PBS before denatured at 100°C for 10 min. After that, 1000 U/mL PNGase F was added at 37°C for more than 5 h following the manufacturer's protocols. The products were then subsequently denatured in the loading buffer and separated by SDS-PAGE. To inhibit the proteasomal degradation, cells were cultured with 10 *μ*M MG132 for different periods of time. To inhibit the lysosomal, cells were cultured with 50 *μ*M CQ for different periods of time.

### 2.3. Western Blot

Western blot analysis was applied as describing in our earlier reports [28]. Briefly, the cultured cells were harvested and then lysed in buffer containing 1% Triton X-100, 20 mM Tris (pH 7.5), 150 mM NaCl, EDTA, leupeptin, sodium pyrophosphate, and phosphatase inhibitors. Cell debris was discarded by centrifugation at 15,000 g for 15 min. Concentrations of the collected proteins were then measured by the Pierce BCA protein assay kit (Thermo Fisher Scientific, shanghai, China). After that, proteins were adjusted to equal amounts for the running of 6% or 10% SDS-PAGE gels and then transferred onto the PVDF membranes (Millipore). After being blocked by 5% TBST containing milk, the membranes were incubated with primary antibodies for target proteins and followed by corresponding secondary antibodies. ECL (Thermo Fisher, Shanghai, China) was used for the finally imaging response.

### 2.4. Plasmid Construction, Site-Directed Mutagenesis, and Transfection

Human full-length SND1 was amplified by PCR using the following primers: 5′-TACCGGACTCAGATCTCGAGCGCCACCATGGCGTCCTCCGCGCAGAGCGGCGGC-3′ and 5′-GATCCCGGGCCCGCGGTACCGTGCGGCTGTAGCCAAATTCGTCTGCATC-3′. The products were then subcloned into GV230 vector (GeneChem Co.) to express SND1-EGFP fusion protein after being digested with XhoI and KpnI. Based on the full full-length SND1 plasmid as the template, four N-glycosylation site mutants for SND1, which replaced the potential sites of asparagine (Asn, N) residues into glutamine (Glu, Q), were constructed. DNA sequencing analysis was utilized to verify the construction.

### 2.5. Construction of Stable Cell Lines

U87 cells were cultured in 6-well plates until 80% confluences. To obtain stable expressing cell lines, fusion plasmids with EGFP expressing wild-type SND1 or four mutants were transiently transfected by Lipofectamine 2000 (Invitrogen, CA, USA) following the manufacturer's instruction. After being transfected for 48 h, the culture medium was added with 1 mg/mL of G418. The dead cells were washed out, and the G418-containing medium was replaced every 2-3 days until only transfected cells with fluorescence signals are left.

### 2.6. Immunofluorescence Staining Assay

Cells were cultured in the chambered cover slips to 30% concentration. After being fixed with 4% paraformaldehyde, cells were permeabilized by 0.5% Triton X-100 and then incubated with 5% BSA for 1 h. Cells with primary antibody (GFP/CNX/GM130) were incubated at 1 : 100 overnight at 4°C. Secondary antibody was then incubated after washed by PBS for 1 hour at room temperature and counterstained with 1 *μ*g/mL DAPI (Beyotime Biotechnology, Shanghai, China) for 10 min, and the cells were mounted by a drop of glycerol-based mounting medium. Confocal laser scanning microscopy (Leica) was utilized to observe fluorescence imaging.

### 2.7. Cell Counting Kit-8 (CCK-8) Assay

Cells were inoculated in the 96-well plates and permitted to grow for approximately 8 h before proceeding with the CCK-8 assay to detect the cell viability. Each well contains 100 *μ*L culture medium. 10 *μ*L CCK-8 solutions were added per well and incubated in the 37°C for another 1.5 h. The optical density (OD) was recorded at 450 nm on the microplate reader. The cell viability was calculated according to the manufacturer's instructions.

### 2.8. Transwell Assay

The transwell inserts were precovered by 100 *μ*L Matrigel (300 *μ*g/mL, Corning Incorporated, Corning, NY, USA). Cells were detached from the culture plates by 0.25% trypsin-EDTA after being cultured into suitable concentration. The culture media were aspirated, the cells in a serum-free DMEM were resuspended, and they were placed into the upper chamber of transwell inserts (8.0 mm, BD Biosciences, Franklin Lakes, NJ) plated in a 24-well plate. 500 *μ*L of complete medium was added into each bottom of the lower chambers in the 24-well plate without moving the transwell insert to avoid the generation of bubbles. After being cultured in 37°C for 8 h, transwell inserts were taken out. Carefully withdraw the upper culture media, and clean the filter side of the upper chambers by cotton-tipped applicators. Then, fix the migrated cells in the reverse sides by 4% paraformaldehyde. Gently wash the migrated cells on the bottom sides by PBS for three times, and then stain them with crystal violet. Migrated cells were viewed and imaged by a phase-contrast microscope. The numbers in different fields were counted and get an average sum of cells.

### 2.9. Statistical Analysis

Statistical analyses were performed by using one way ANOVA with the GraphPad Prism 6 software. Results are expressed as the means ± standard error of the mean (SEM). Statistical significance was admitted at ^∗^*p* < 0.05 and ^∗∗^*p* < 0.01. All the experiments were repeated at least three times.

## 3. Results

### 3.1. High Expression of SND1 Is Related to Poor Survival of Glioma Patients

**Figure 1:**
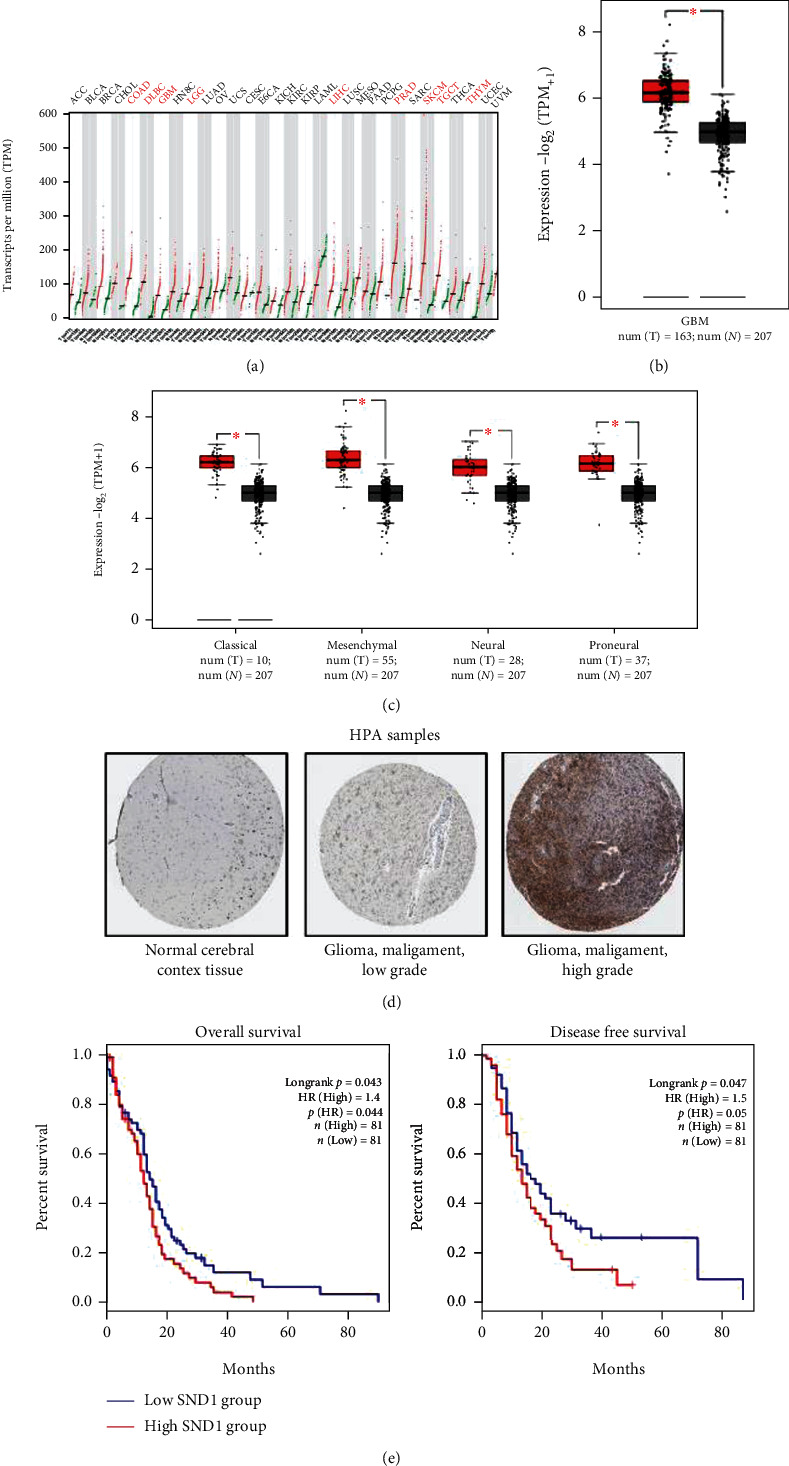
Expression level of SND1 gene in different cancers and pathological stages. The expression status of the human SND1 gene in different cancers tissues (a), (b) GBM, or specific GBM subtypes (c) was analyzed through GEPIA 2 (http://gepia2.cancer-pku.cn/#analysis). (d) Immunohistochemical staining of SND1 in clinical glioma tissue samples and normal brain tissues were obtained from the Human Protein Atlas (http://www.proteinatlas.org). Normal tissue, access numbers: NOS (M-00100), patient ID: 1582; glioma, malignant: low grade (M-938031), Patient ID: 3120; glioma, malignant: high grade (M-938033), patient ID: 46. (e) The effect of SND1 on the overall survival and disease-free survival of glioblastoma patients was acquired from GEPIA 2.

To determine the SND1 expression levels in different cancers, the GEPIA 2 (http://gepia2.cancer-pku.cn/#index) database was applied. The results suggested that SND1 was significantly overexpressed in various cancer tissues than that in the corresponding normal tissues, including colon adenocarcinoma (COAD), lymphoid neoplasm diffuse large B-cell lymphoma (DLBC), liver hepatocellular carcinoma (LIHC), prostate adenocarcinoma (PRAD), skin cutaneous melanoma (SKCM), testicular germ cell tumors (TGCT), thymoma (THYM), glioblastoma (GBM), and lower grade glioma (LGG). The upregulation of SND1 in GBM was seemed higher than that in LGG (Figure 1(a)). We further evaluated the difference of SND1 between the GBM and normal tissues by matching TCGA and GTEx data; SND1 was obviously highly expressed in GBM patients (Figure 1(b)). The results were in line among the four subtypes of GBM (Figure 1(c)). SND1 expression in glioma was also observed in immunohistochemistry, and results showed that SND1 was significantly upregulated in clinical glioma samples, especially in GBM (Figure 1(d)). Analysis of survival data presented that higher SND1 expression was correlated with poor overall survival (OS) (*p* = 0.043) and disease-free survival (DFS) (*p* = 0.047) according to the log-rank tests (Figure 1(e)). Herein, we hypothesize that SND1 might play a critical role in the progression of glioma.

### 3.2. SND1 Is N-Glycosylated in Glioma Cells

**Figure 2:**
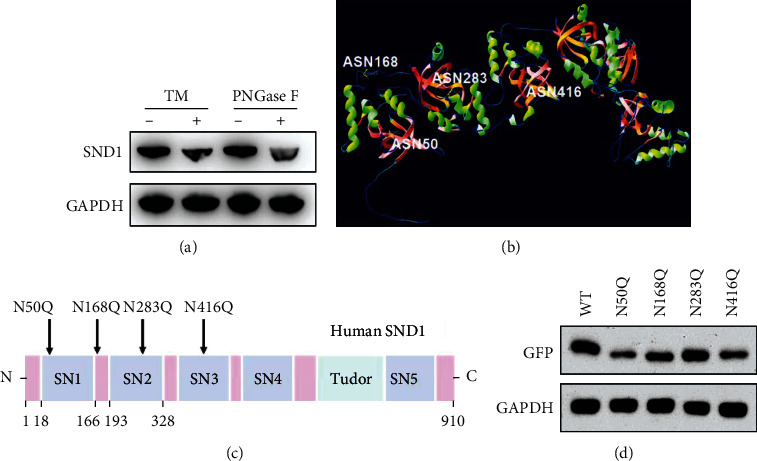
Analysis of N-glycosylation in SND1. (a) Human glioma U87 cells were treated by tunicamycin (TM) and peptide -N-glycosidase F (PNGase F); the level of SND1 was determined by electrophoresis in 6% SDS-PAGE and then immunoblotted with anti-SND1 antibodies. (b) Three-dimensional structure for human SND1. Locations of four potential N-glycosylation sites (Asn50, Asn168, Asn283, and Asn416) were indicated in yellow. (c) The schematic illustrates structural domains of human SND1 and its potential N-glycosylation site mutagenesis (N50Q, N168Q, N283Q, and N416Q) SN: staphylococcal nuclease domains. Asn (N); Gln (Q). (d) Cell lysate from U87 cells expressing WT-SND1, or its single mutants were analyzed by 6% SDS-PAGE with an anti-GFP antibody. GAPDH was used as a loading control.

Consider the critical role of SND1 in glioma; we firstly detected the presence of N-glycosylation for SND1 in glioma cells. U87 cells were treated with tunicamycin (TM), which blocks the N-glycosylation by inhibiting the core oligosaccharide attached to the nascent polypeptides, and the peptide-N-glycosidase F (PNGase F), which functions as an amidase that cleaves almost all types of N-glycans from glycoproteins. A remarkable mobility shift was noted after treatment, suggesting that N-glycans are ubiquitously present on SND1 in glioma cells (Figure 2(a)). Subsequent studies were all carried out in U87 cells. Human SND1 protein sequence consists of 910 amino acids and contains four potential N-glycosylation sites (Asn-Xaa-Ser/Thr) at the Asn50, Asn168, Asn283, and Asn416, which were predicted by the NetNGlyc-1.0 program (https://services.healthtech.dtu.dk/service.php?NetNGlyc-1.0) and shown in three-dimension (Figure 2(b)). To clarify these four N-glycosylation sites that were occupied by N-glycans, we construct four mutant forms for SND1 (N50Q, N168Q, N283Q, and N416Q), replacing the Asn (N) into Gln (Q) (Figure 2(c)). Each of the four mutants showed a significant mobility shift on SDS-PAGE gels compared with the wild type (WT), suggesting that SND1 was heavily N-glycosylated on these four predicted N-glycosylation sites (Figure 2(d)).

### 3.3. Removal of N-Glycans on the Asn50 Site of SND1 Blocks Its ER-to-Golgi Transport

**Figure 3:**
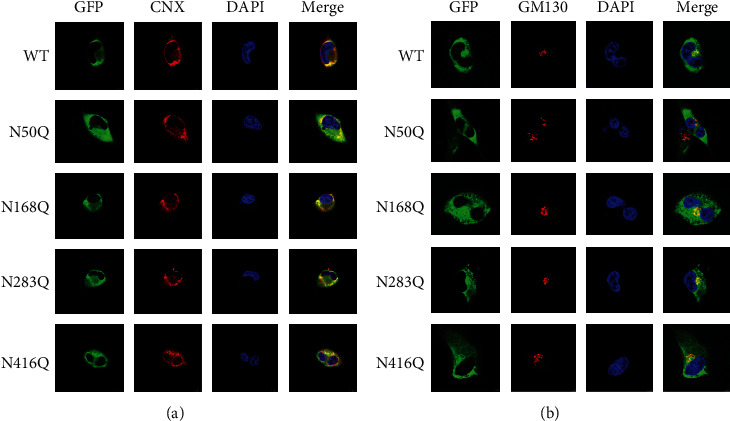
Subcellular localization of SND1 N-glycosylation mutants. (a) The localization of WT-SND1-EGFP and its four N-glycosylation mutants (green), CNX (red), and DAPI (blue) in U87 cells. (b) Immunostaining for WT-SND1-EGFP and its four N-glycosylation mutants (green), GM130 (red), and DAPI (blue) in U87 cells. Scale bar, 10 *μ*m.

Previous study indicated that more than a third of proteins in eukaryotes were targeted to the ER lumen, and then they were modified by glycans through ER to the Golgi and finally to the functional state [10]. When inhibiting the glycosylation, the proteins might be misfolded and retained in the ER and thus failed to reach the destination and be targeted for degradation [10, 29]. To detect the effect of N-glycosylation on SND1 stability, the subcellular localization of SND1 and its four mutants was observed under confocal laser scanning microscopy. Costaining of SND1 and ER marker calnexin (CNX) showed that cells overexpressing WT-SND1 and N168Q, N283Q, and N416Q mutants were partially colocalized with CNX in the cytoplasm, whereas cells overexpressing N50Q showed significantly higher colocalization with CNX than cells overexpressing WT-SND1 and other mutants (Figure 3(a)). In contrast, costaining of SND1 and the Golgi marker GM130 indicated that cells overexpressing N50Q had little colocalization, while the cells overexpressing other mutants as well as WT-SND1 were fixed with GM130 very well. These data suggested that the N50Q mutant might accumulate in the ER and thus block the ER-Golgi transport glioma cells (Figure 3(b)).

### 3.4. Removal of the N-Glycans on the Asn50 Site of SND1 Induces ER-Associated Degradation

**Figure 4:**
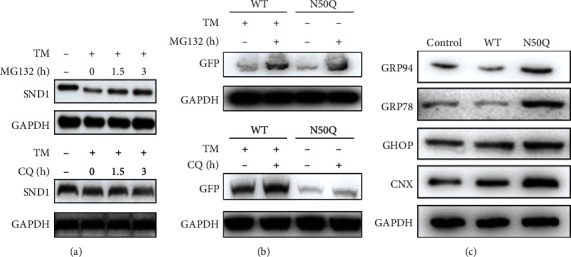
N-glycosylation of Asn50 induces ER-associated degradation of SND1. (a) U87 cells overexpressing wild-type SND1-EGFP were pretreated with 1 *μ*g/mL TM for 24 h, followed by culture with 10 *μ*M MG132 or 50 *μ*M CQ for 0 h, 1.5 h, and 3 h. The expression level of exogenous SND1 was then determined by immunoblot. (b) U87 cells overexpressing wild-type SND1-EGFP and N50Q-EGFP were treated with MG132 for 3 h before collection of whole lysates. Exogenous expression was then determined by immunoblot. (c) Cells expressing EGFP, SND1-EGFP, and N50Q-EGFP were analyzed by western blot to determine the levels of GRP94, GRP78, CHOP, and CNX. GAPDH was used as a loading control. All these data are representative of at least three independent experiments.

To further test the effect of N-glycans on the Asn50 of SND1 for cellular stability, we firstly detected the degradation mode for SND1. After TM treatment for 48 h, the SND1 protein level was markedly downregulated. However, in the condition of MG132, an inhibitor of proteasomal degradation, the protein level of SND1 was gradually reversed by the increase of MG132 concentration. We also treated the cells with chloroquine (CQ), an inhibitor of lysosomal degradation, and no discernible difference could be observed (Figure 4(a)). To investigate whether N-glycans on the Asn50 have a similar effect on SND1, comparative analysis was then performed between the TM-treated WT-SND1 and N50Q overexpression cell lines. Results revealed that the degradation of SND1 mediated by N50Q mutation could be partially reversed by MG132, but not CQ (Figure 4(b)). These results suggested that the removal of N-glycans on Asn50 might destabilize SND1 expression through the proteasomal degradation pathway. Taking together the above findings, N50Q-expressing cells induced an ER accumulation and proteasome-based degradation, which are consistent with typical characteristics of endoplasmic reticulum-associated degradation (ERAD) substrates. According to the previous study, proteins are folded correctly under the assistance of ER chaperones before they transfer to the Golgi, and unfolded or/and misfolded proteins that retain in the ER would be retrogradely transported into the cytoplasm by the ERAD and then degraded by proteasomes. The folding process and ERAD would be kept in a balance. High accumulation of incorrect folded proteins would activate the ER stress and then trigger unfolded protein response (UPR) [30, 31]. In our study, we found significantly increased in the expression of ER chaperones that assist in protein folding and force the incorrect folded proteins to ERAD including GRP94 (HSP90B1) and GRP78 (HSPA5, Bip) (Figure 4(c)). UPR was also activated in N50Q overexpressing cells, as shown in the increasing levels of CHOP and CNX, which are both markers that upregulated in the ER stress (Figure 4(c)). These results suggested that N50Q-overexpressing cells might be unable to fold SND1 in a proper manner, thus triggered the ER stress response, attracted the chaperones to induce ERAD, and finally degraded the N50Q by proteasomal.

### 3.5. Removal of N-Glycans on the Asn50 Site of SND1 Suppresses the Cell Proliferation and Metastasis

**Figure 5:**
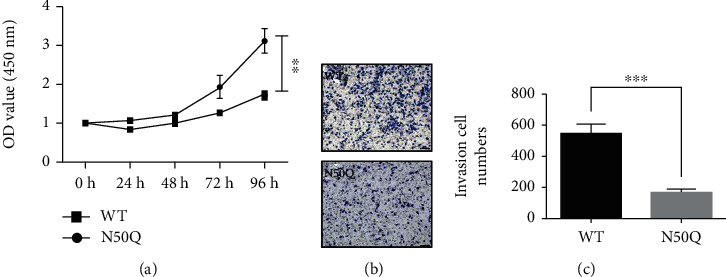
Elimination of N-glycans at Asn50 inhibits cell proliferation and metastasis. (a) The proliferation ability of glioma cells was detected by CCK8 assay performed on both wild-type SND1-EGFP and N50Q-EGFP overexpressing cells with a duration of 4 days. (b) The cell invasion assay was detected by transwell assay administrated in the wild-type SND1-EGFP and N50Q-EGFP overexpressing cells, and invaded cells were fixed and stained with 0.5% crystal violet. Scale bar: 100 *μ*m. (c) Representative images of the bottom surface were captured using a phase-contrast microscopy (upper). The numbers of migrated cells in nine random microscopic fields (×200) were counted and averaged. Data represent the mean ± SEM of three independent experiments. ^∗^*p* < 0.01; ^∗∗^*p* < 0.001.

To further determine whether the N-glycosylation-regulated control of SND1 stability has effects on the oncogenicity in glioma, we investigated the cell proliferation and cell metastasis in glioma cells. Results indicated that removal of N-glycans on the Asn50 of SND1 significantly suppressed the cell proliferation compared with the WT-SND1 overexpression cells (Figure 5(a)). Then, we used the transwell assay to evaluate the migratory ability of glioma cells. A markedly decrease in the number of metastasis cells were observed in the N50Q stably expressing cells compared with WT-SND1 control expressing cells (Figures 5(b) and 5(c)).

## 4. Discussion

Human SND1 is an evolutionarily conserved protein that encodes 910 amino acids and comprises tandem repeats SN1-4 and a fusion TSN domain [32]. The simultaneous existence in domains made the SND1 a highly protein-protein or protein-RNA binding affinity. Thus, it can function as both a nuclease or/and a scaffolding member of multiprotein complexes. Initial studies discovered SND1 as a transcriptional coactivator. Subsequently, SND1 was demonstrated to play roles in posttranscriptional regulation, such as splicing mRNA and RNAi. Recently, SND1 has gained attention due to its high expression of protein level in several cancers. Upregulation or/and function activation of SND1 was considered to positively associated with cancer progression [33]. In the current study, we provided insights on the functions of N-glycosylated SND1 in glioma. SND1 was overexpressed in both LGG and GBM compared with the healthy. The upregulation of SND1 expression significantly correlated with glioma grades and poor prognosis. To our interest, the mechanisms of SND1 in glioma were not fully understood.

It is well accepted that glycosylation is the most abundant modification after translation of proteins and is associated with multiple cellular events, such as inflammation, carcinogenesis, and cancer metastasis [34, 35]. Alteration of glycosylation is now regarded as a feature hallmark in several cancer progression [36]. Most proteins in the eukaryotic cells undergo the glycosylation process, so as the SND1. Human SND1 is predicted to contain four potential N-glycosylation sites (Asn50, Asn168, Asn283, and Asn416), characterized by the specific Asn-Xaa-Ser/Thr motif. Three of them are located in the SN function domains and one of them is located in the invariant region. In this study, we suggested that all the four potential N-glycosylation sites of SND1 were occupied by the N-glycans in glioma cells using site-directed mutagenesis.

Most of the glycosyltransferases essential for the glycosylation process contain catalytic domains orient the ER and Golgi. The transfer of initial glycans onto the glycoproteins usually starts in the ER or on the membrane of ER [37]. Subsequently, proteins are further added with several sugars under the catalyzation of glycosyltransferases to mature, are correctly folded for transit to the Golgi, and finally reached the destinations and function [10]. SND1 was also reported to be anchored on the membrane of ER and function as an ER-associated protein [38]. Thus, we checked the colocalization of SND1 with ER marker. Confocal microscopy images showed that by removing the N-glycans on Asn50, SND1 was predominantly retained in the ER, but this phenomenon was not observed in the N168Q-, N283Q-, and N416Q-overexpressing cells. The removal of glycans in the N-terminal of glycoproteins was reported to be more sensitive, since the protein folding machinery always initiates from the N-terminal to force protein folding correctly [39]. Our results above seem to confirm this conclusion since Asn50 was the N-glycosylation site mostly near the N-terminal.

When the protein glycosylation was inhibited, the most often observed was the generation of numerous aggregated, misfolded, or/and unfolded proteins [10]. The generated misfolded or/and unfolded proteins would then be sent back to refold or tagged to degradation through ERAD. We also checked the colocalization of SND1 with a Golgi marker. Results suggested less of N50Q was expressed in the Golgi apparatus, which induced us to further study whether the N50Q of SND1 was unfolded or misfolded in the ER and thus failed to be transported to Golgi apparatus, and then selectively eliminated via the ER-associated degradation.

When the unfolded or misfolded proteins accumulate too much in the ER lumen, the specific signaling, UPR, would be triggered. UPR was initially mentioned upon the upregulation of glucose-regulated proteins (GRPs) [40]. Among the GRPs, GRP78 was most abundant and the first one to be identified as an ER molecular chaperone [41, 42].

Subsequently, another ER molecular chaperone, GRP94, was also demonstrated to upregulate in the UPR [43]. Activation of ER stress by the continuous stimulus should be the most upstream for triggering UPR. The stimulus should be numerous unfolded proteins [44]. Current study suggested that the GRP78 and GRP94 were both upregulated in the N50Q cells, indicating that removal of the N-glycans on the Asn50 induced a UPR under ER stress. Meanwhile, CHOP, which indicates overexpressed misfolded proteins reduced the prolonged UPR as well as the CNX, which is required in the correct folding as a lectin chaperones, was also increased in the N50Q overexpression cells.

The cell state with an overload of numerous incorrect proteins in the ER also called ER stress starts up a self-regulated ERAD to protect cells from UPR activation [45]. This phenomenon also helps to explain why mutant Asn50, the expression of SND1was decreased, and the decrease could be reversed by the MG132. The answer might be this: removal of the N-glycans on the Ans50 disrupts the correct manner to fold proteins, and the incorrectly folded proteins might accumulate too much in the cells and lead to the ERAD through proteasomes.

In addition, sustained ER stress activates UPR that also damages the cellular homeostasis.

The results of breaking this homeostasis in different malignancies might induce the occurrence of oxidative stress response, autophagy, and inflammatory response [46]. All these reactions are thought to be related to the change of cancer progress. Our results suggested the N50Q mutant inhibits both cell proliferation and metastasis in glioma, which may be also associated with the incorrectly folded protein-induced homeostasis damage.

In summary, our study identified the four N-glycosylation sites in SND1. And we suggested that N50Q in the N-terminal SN domain of SND1 is essential for the maintenance of protein stability. Remarkably, we postulated that N50Q could not fold correctly and block the ER-to-Golgi transport. The following state of ER stress and UPR was degraded by proteasomes in an ER-associated manner. Besides, we demonstrated that N-glycosylation on the Asn50 is important for the cell proliferation and metastasis.

## Data Availability

The data that support the findings of this study are available from the corresponding authors upon request.
